# Complete Genome Sequence of a Natural Recombinant H9N2 Influenza Virus from Wild Birds in Republic of Korea

**DOI:** 10.1128/genomeA.00159-12

**Published:** 2013-02-14

**Authors:** Dong-Hun Lee, Jae-Keun Park, Seong-Su Yuk, Tseren-Ochir Erdene-Ochir, Jung-Hoon Kwon, Joong-Bok Lee, Seung-Yong Park, In-Soo Choi, Chang-Seon Song

**Affiliations:** Avian Disease Laboratory, College of Veterinary Medicine, Konkuk University, 1 Hwayang-dong Gwangjin-gu, Seoul, Republic of Korea

## Abstract

We report the complete genome sequence of a natural recombinant H9N2 avian influenza virus (AIV) that was isolated from a wild bird in the Republic of Korea in 2005. The genomic sequence and phylogenetic analyses showed that this virus contains six genes of North American lineage AIV and two genes of Eurasian lineage AIV. These data are beneficial for investigating the ecology and epidemiology of AIVs.

## GENOME ANNOUNCEMENT

Avian influenza virus (AIV) belongs to the influenza virus A genus of the *Orthomyxoviridae* family (1). The AIV genome consists of eight segments of negative-sense, single-stranded RNA that encode at least 10 proteins, including 2 surface glycoproteins (hemagglutinin [HA] and neuraminidase [NA]), nucleoprotein (NP), three polymerase proteins (polymerase basic 2 [PB2], PB1, and polymerase acidic [PA]), two matrix (M) proteins (M1 and M2), and two nonstructural (NS) proteins (NS1 and NS2). AIVs are classified into subtypes based on the antigenic differences between their two surface glycoproteins, hemagglutinin (HA) and neuraminidase (NA). Sixteen HA subtypes (H1 to H16) and nine NA subtypes (N1 to N9) have been identified among influenza A viruses, and viruses of all subtypes and of the majority of possible combinations have been isolated from avian species (1). Phylogenetic analysis has revealed two geographically separate AIV clades, North American and Eurasian, despite occasional intercontinental virus exchange in overlapping bird migration routes (2, 3).

In the present study, an H9N2 strain, named A/wild bird/Korea/8g-39/2005(H9N2), was isolated from a wild bird in the Republic of Korea in 2005. The complete genome of the virus was amplified by reverse transcription PCR and sequenced with an ABI PRISM 3730xl genetic analyzer (Applied Biosystems, Foster City, CA). The results indicated that the full lengths of each segment (PB2, PB1, PA, HA, NP, NA, M, and NS) were 2,309, 2,309, 2,208, 1,683, 1,502, 1,432, 999, and 861 nucleotides, respectively. The eight genes encoded the following proteins and their deduced amino acid lengths: PB2, 759; PB1, 757; PB1-F2, 90; PA, 716; HA, 560; NP, 498; NA, 469; M1, 252; M2, 97; NS1, 230; and NS2, 121. The deduced amino acids at the cleavage site of the HA protein were PAASDR↓GLF, with the characteristic property of low pathogenic potential.

Phylogenetic analysis revealed that the PB2, PA, HA, NP, NA, and NS genes of the A/wild bird/Korea/8g-39/2005(H9N2) virus were derived from North American lineages, but the PB1 and M genes were derived from Eurasian lineages. It seems that the coinfected wild bird population allows genes from different lineages to mix, which results in reassortant North American and Eurasian lineage AIV.

In this study, our results provide important information on the ecology and epidemiology of avian influenza viruses, defining the role of wild birds in transferring AIV between the Eurasian and American continents. Continued surveillance of wild bird populations could significantly improve our understanding of the role of migratory birds in the natural history of AIV.

### Nucleotide sequence accession numbers.

The genome sequences of A/wild bird/Korea/8g-39/2005(H9N2) have been deposited in GenBank under accession numbers KC209494 to KC209501.
